# Pre-treatment microbial *Prevotella*-to-*Bacteroides* ratio, determines body fat loss success during a 6-month randomized controlled diet intervention

**DOI:** 10.1038/ijo.2017.220

**Published:** 2017-10-10

**Authors:** M F Hjorth, H M Roager, T M Larsen, S K Poulsen, T R Licht, M I Bahl, Y Zohar, A Astrup

**Affiliations:** 1 Department of Nutrition, Exercise and Sports, Faculty of Science, University of Copenhagen, Frederiksberg, Denmark; 2 National Food Institute, Technical University of Denmark, Kgs. Lyngby, Denmark; 3 Steno Diabetes Center Copenhagen, Gentofte, Denmark; 4 Gelesis Inc., Boston, MA, USA

## Abstract

On the basis of the abundance of specific bacterial genera, the human gut microbiota can be divided into two relatively stable groups that might have a role in personalized nutrition. We studied these simplified enterotypes as prognostic markers for successful body fat loss on two different diets. A total of 62 participants with increased waist circumference were randomly assigned to receive an *ad libitum* New Nordic Diet (NND) high in fiber/whole grain or an Average Danish Diet for 26 weeks. Participants were grouped into two discrete enterotypes by their relative abundance of *Prevotella* spp. divided by *Bacteroides* spp. (*P/B* ratio) obtained by quantitative PCR analysis. Modifications of dietary effects of pre-treatment *P/B* group were examined by linear mixed models. Among individuals with high *P/B* the NND resulted in a 3.15 kg (95% confidence interval (CI): 1.55; 4.76, *P*<0.001) larger body fat loss compared with ADD, whereas no differences was observed among individuals with low *P/B* (0.88 kg (95% CI: −0.61; 2.37, *P*=0.25)). Consequently, a 2.27 kg (95% CI: 0.09; 4.45, *P*=0.041) difference in responsiveness to the diets were found between the two groups. In summary, subjects with high *P/B* ratio appeared more susceptible to lose body fat on diets high in fiber and whole grain than subjects with a low *P/B* ratio.

## Introduction

The composition of the gut microbiota in rodents has been shown to affect the efficacy of energy harvest from feed^1^ and to influence the secretion of gastrointestinal hormones affecting appetite.^2^ Therefore, it seems as if the human gut microbiota has the potential to have a pivotal role in personalized nutrition.^3, 4^

Clustering of the human gut microbiota, designated enterotypes, was first described in 2011.^5^ The *Bacteroides*-driven enterotype is reported to be predominant in individuals consuming more protein and animal fat (western diet), whereas the *Prevotella-*driven enterotype appears predominant in subjects consuming more carbohydrate and fiber.^6, 7, 8^ That said, the enterotype of an individual has been shown to remain rather stable.^6, 7, 9^ A limited number of studies have related microbial enterotypes to health markers;^8, 9, 10^ however, body fat change during a randomized clinical trial is not one of them.

Therefore, as a proxy for enterotypes, we studied pre-treatment *Prevotella*-to-*Bacteroides (P/B)* ratio as a prognostic marker for successful body fat loss on two diets differing greatly in dietary fiber and whole-grain content.

## Materials and methods

In total 181 participants with increased waist circumference were randomly assigned to receive an *ad libitum* New Nordic Diet (NND) or a control diet for 26 weeks of which a subgroup of 62 subjects were randomized to collect fecal samples. The macronutrient composition of the NND was based on Nordic Nutrition Recommendations, whereas the control diet was designed to match the macronutrient composition of an Average Danish Diet (ADD).^11^ The NND is a whole-food approach characterized by being very high in dietary fiber, whole grain, fruit and vegetables.^12^ For both groups, food and beverages were provided from a study shop free of charge throughout the intervention period.^12^ Pre-intervention fasting blood samples were drawn from where fasting glucose and insulin were analyzed. Height was measured at baseline and body weight was measured at randomization and week 2, 4, 8, 12, 16, 20, 24 and 26. Furthermore, waist circumference and fat mass (using DEXA) were measured at randomization, week 12 and 26. Fecal samples were collected at baseline and the relative abundance of *Prevotella* spp. and *Bacteroides* spp. was determined using genera-specific quantitative PCR targeting the bacterial 16S ribosomal gene regions as previously described.^9^ As previously reported by Roager *et al.*,^9^ this resulted in a clear bimodal separation of subjects based on the log *Prevotella* spp. to *Bacteroides* spp. ratio, in the following designated low *P/B* (<0.01) or high *P/B* (>0.01). In eight samples, *Prevotella* spp. was below the detection limit and were classified as low *P/B* in the main analysis and excluded in a sensitivity analysis. Regardless of randomization status, after the completion of the first 26 weeks, all participants were instructed to follow the NND for an additional year (weight measured after 52 and 78 weeks) without any provision of food^13^ to investigate the diets in a real life setting. The study was approved by the ethical committee of the Capital Region of Denmark (reference H-3-2010-058) and registered at clinicaltrials.gov as NCT01195610.

### Statistics

Baseline characteristics were summarized as mean±s.d., median (interquartile range) or proportions (%) and differences between *P/B* groups as well as dietary groups were tested using a parametric (some variables transformed before analysis) or non-parametric two-sample test or Pearson’s *χ*^2^ test.

The differences in body fat (as well as weight and waist circumference) change from baseline between P/B groups on the two diets were analyzed by means of linear mixed models using all available measurements. The linear mixed models included the three-way interaction between diet × time × *P/B* group strata as well as all nested two-way interactions and main effects and comprised additional fixed effects including age, gender, baseline BMI, baseline fasting glucose and insulin as well as random effects for subjects. Results are shown as mean change from baseline with 95% confidence interval (CI). The level of significance was set at *P*<0.05 and statistical analyses were conducted using STATA/SE 14.1 (Houston, TX, USA).

## Results

The NND compared to ADD was higher in dietary fiber (43.3 vs 28.6 g/10MJ), higher in protein (18.1 vs 16.4%), lower in fat (30.4 vs 33.8%) (all *P*<0.001) without differing in available carbohydrates (46.4 vs 45.3% *P*=0.081).

No differences in baseline characteristics were found between individuals characterized as high and low *P/B* (all *P*⩾0.09) (Table 1). Among individuals with a high *P/B* ratio, the NND diet resulted in a 3.15 kg (95% CI: 1.55; 4.76, *P*<0.001) larger body fat loss compared to ADD after 26 weeks, whereas no difference in body fat loss was observed between NND and ADD among individuals with low *P/B* (0.88 kg (95% CI: −0.61; 2.37, *P*=0.25)). Consequently, a 2.27 kg (95% CI: 0.09; 4.45, *P*=0.041) difference in responsiveness to the diets was found between the *P/B* groups, which came from difference in response to NND (*P*=0.04) and not ADD (*P*=0.41) between the *P/B* groups (Table 2). Similar differences in responsiveness to the diets were found for waist circumference (3.95 cm (95% CI: 0.34; 7.55, *P*=0.032)) and were borderline significant for body weight (2.33 kg (95% CI: −0.15; 4.80, *P*=0.065)) (Table 2). The sensitivity analysis revealed larger differences (Table 2).

During the 1 year follow-up period, subjects with the high *P/B* ratio changing from ADD to being recommended NND maintained their weight (−1.23 (95% CI: −2.81; 0.36, *n*=9, *P*=0.13)), whereas subjects with the low *P/B* ratio changing from ADD to being recommended NND regained 2.76 kg (95% CI: 1.27; 4.24, *n*=11, *P*<0.001). Consequently, a 3.99 kg (95% CI: 1.82; 6.15, *P*<0.001) difference in responsiveness to the NND were found between *P/B* groups during the 1 year follow-up. This difference was 5.41 kg (95% CI: 3.12; 7.69, *P*<0.001) in the sensitivity analysis.

## Discussion

We identified pre-treatment *P/B* ratio as an important biomarker associated with body fat loss in subjects consuming an *ad libitum* diet rich in fiber and whole grain. Thus, overweight and obese participants with high *P/B* ratio appeared more responsive to fiber and whole grain than individuals with low *P/B* ratio. This was further supported by similar findings for waist circumference and body weight.

Using the entire sample of 181 subjects, we have previously reported the overall weight-loss difference between the NND and ADD to be 3.2 kg.^12^ Interestingly, this difference between diets could mainly be attributed to subjects with the high *P/B* ratio, and the health-promoting aspects of the NND in terms of body-weight regulation, therefore, mainly seems to apply in a subset of the population.

Previously, baseline total cholesterol has been found to be borderline higher (*P*=0.08)^9^ and LDL cholesterol to be lower^8^ among the Prevotella-driven enterotype. Furthermore, the enterotypes have been found to impact *in vitro* fermentation profiles of short chain fatty acids from the same carbohydrate substrates differentially, with the *Prevotella*-driven enterotype having higher total short chain fatty acid production.^3^ *In vitro*, some of these short chain fatty acids have been shown to stimulate the secretions of gastrointestinal hormones affecting appetite.^2^ Finally, in an observational study of 1632 women, the abundance of *Bacteroides* spp. was associated with weight gain, whereas dietary fiber intake was found partly to modify the association between microbiome diversity and weight gain.^14^

The distinction of enterotypes as discrete clusters has recently been challenged by studies suggesting that enterotype distribution is continuous and that further information may be masked within these enterotype clusters.^15, 16^ From our analysis, we cannot determine specific bacterial species responsible for the dietary effects that we observe but only highlight the relative abundance of *Prevotella* spp. (genus) as important in the classification of microbiota profiles. Nevertheless, our sensitivity analysis indicates that subjects with *Prevotella* spp. below the detection limit behave different than subjects in the low *P/B* ratio group.

The increased responsiveness of the high *P/B* group to the NND, rich in fruits, vegetables, dietary fibers and whole grains, is supported by previous studies showing an association between the *Prevotella*-driven enterotype and a carbohydrate-based diet more typical of agrarian societies.^6^ However, only two individuals switched *P/B* ratio group during this 6-month dietary intervention with NND or ADD,^9^ which is consistent with the literature indicating that intestinal microbial communities are resilient and difficult to change through dietary interventions^6, 7, 9^ unless extreme changes, such as complete removal of carbohydrates from the diet, are introduced.^17^

Mechanisms involved could be efficacy of energy harvest from different foods,^1^ differences in fiber-utilization capacity,^3^ gut-brain signaling of behavior^18^ and the secretion of gastrointestinal hormones affecting appetite.^2, 10^ Recently, dietary fiber-induced improvements in post-prandial blood glucose and insulin were found to be positively associated with the abundance of *Prevotella*.^19^ Therefore, the recent breakthrough in personalized nutrition, showing the importance of pre-treatment fasting glucose and insulin to determine the optimal diet for weight management,^20^ might also be linked to gut microbiota profiles. We therefore adjusted for a number of potential confounders including fasting glucose and insulin. However, independent of the mechanisms, the *P/B* ratio may serve as a biomarker to predict future weight-loss success on specific diets.

In summary, we identified pre-treatment *P/B* ratio as an important biomarker associated with dietary body fat change on *ad libitum* high fiber diets. Thus, individuals with a high *P/B* ratio were more susceptible to body fat loss on a diet rich in fiber and whole grain compared to an average Danish diet, whereas no difference in body fat loss was observed in individuals with a low *P/B* ratio.

## Figures and Tables

**Table 1:** Baseline characteristics of the study populations stratified by enterotype (*n*=62)

	*High* P/B *group (*n=*28)*	*Low* P/B *group (*n=*34)*	P*-value*
Age (year)	41.9 (30.4; 56.7)	47.5 (33; 55.6)	0.33
Gender (%female/male)	64.3/35.7	69.2/30.8	0.70
Body weight (kg)	91.6±17.6	84.8±16	0.12
Body mass index (kg m^−2^)	31.0±4.7	29.0±4.4	0.09
Body fat (%)	40.5±6.4	38.9±7.1	0.36
Fasting glucose (mmol l^−1^)	5.34±0.51	5.19±0.40	0.20
Fasting insulin (pmol l^−1^)	54.5 (41; 78)	47.5 (35; 74)	0.14
*Prevotella* spp. (relative abundance)	0.016 (0.008; 0.063)	0.00002 (0.000003; 0.00005)	<0.001^a^
*Bacteroides* (relative abundance)	0.07 (0.05; 0.11)	0.17 (0.10; 0.26)	<0.001^a^
*Prevotella*-to-*Bacteroides* ratio	0.28 (0.11; 7.50)	0.00007 (0.00001; 0.00026)	

Abbreviation: *P/B*, *Prevotella*-to-*Bacteroides*.

a Using the non-parametric two-sample Wilcoxon rank-sum (Mann–Whitney) test.

Data are presented as mean±s.d., median (interquartile range) or proportions (%) and differences between enterotypes were tested using a two-sample *t*-test (variables possibly transformed before analysis) or Pearson’s *χ*^2^ test.

**Table 2:** Changes in body fat, body weight and waist circumference after 26 weeks on NND and ADD among high *P/B* and low *P/B* groups

	*High* P/B *group*		*Low* P/B *group*		P^a^	P^b^	P^c^	P^d^	*Δ(NND-ADD) in high* P/B *−Δ(NND-ADD) in low* P/B	P^e^
*All subjects*	NND (*n*=15)	ADD (*n*=13)	NND (*n*=21)	ADD (*n*=13)						
ΔBody fat (kg)	−4.97 (−6.06; −3.88)	−1.82 (−3.01; −0.63)	−3.41 (−4.35; −2.48)	−2.53 (−3.69; −1.37)	<0.001	0.25	0.04	0.41	−2.27 (−4.45; −0.09)	0.041
ΔWeight (kg)	−4.58 (−5.82; −3.34)	−1.09 (−2.43; 0.25)	−3.27 (−4.33; −2.22)	−2.11 (−3.43; −0.79)	<0.001	0.18	0.12	0.29	−2.33 (−4.80; 0.15)	0.065
ΔWC (cm)	−5.19 (−6.99; −3.38)	−0.44 (−2.41; 1.52)	−3.09 (−4.64; −1.55)	−2.29 (−4.22; −0.37)	<0.001	0.53	0.09	0.19	−3.95 (−7.55; −0.34)	0.032
										
*Sensitivityf*	NND (*n*=15)	ADD (*n*=13)	NND (*n*=16)	ADD (*n*=10)						
ΔBody fat (kg)	−4.96 (−5.95; −3.97)	−1.79 (−2.87; −0.71)	−2.94 (−3.93; −1.94)	−2.71 (−3.92; −1.50)	<0.001	0.78	0.01	0.27	−2.94 (−5.05; −0.85)	0.006
ΔWeight (kg)	−4.57 (−5.70; −3.45)	−1.07 (−2.29; 0.15)	−2.52 (−3.64; −1.40)	−2.56 (−3.93; −1.18)	<0.001	0.97	0.01	0.12	−3.53 (−5.92; −1.15)	0.004
ΔWC (cm)	−5.14 (−6.91; −3.36)	−0.54 (−2.47; 1.39)	−2.29 (−4.07; −0.52)	−3.60 (−5.76; −1.43)	<0.001	0.36	0.03	0.04	−5.90 (−9.65; −2.14)	0.002

Abbreviations: ADD, Average Danish Diet; NND, New Nordic Diet; *P/B*, *Prevotella*-to-*Bacteroides*; WC, Waist circumference.

Data are presented as estimated mean body fat, body weight and waist circumference change from baseline and 95% confidence intervals for each combination of the diet-P/B strata interaction after 26 weeks in the linear mixed models, which were additionally adjusted for age, gender, baseline BMI, fasting glucose and insulin as well as random effects for subjects.

a *P*-value representing the difference in dietary response within the high *P/B* group.

b *P*-value representing the difference in dietary response within the low *P*/B group.

c *P*-value representing the difference in response to NND between the *P/B* groups.

d *P*-value representing the difference in response to ADD between the *P/B* groups.

e *P*-value representing the following pairwise comparison using *post hoc t*-tests: Δ(NND-ADD) among subjects with high *P/B*−Δ(NND-ADD) among subjects with low *P/B*.

f Sensitivity analyses excluding the eight subjects with *Prevotella* spp. below the detection limit.
